# Correction: Pru p 9, a new allergen eliciting respiratory symptoms in subjects sensitized to peach tree pollen

**DOI:** 10.1371/journal.pone.0232301

**Published:** 2020-04-21

**Authors:** Miguel Blanca, Laura Victorio Puche, María Garrido-Arandia, Laura Martin-Pedraza, Alejandro Romero Sahagún, José Damian López-Sánchez, Carmen Galán, Antonio Marin, Mayte Villalba, Araceli Díaz-Perales, Maria Luisa Somoza

The ninth author's name is spelled incorrectly. The correct name is: Mayte Villalba.
