# Correction: Pru p 9, a new allergen eliciting respiratory symptoms in subjects sensitized to peach tree pollen

**DOI:** 10.1371/journal.pone.0314603

**Published:** 2024-11-21

**Authors:** Natalia Perez-Sanchez, Miguel Blanca, Laura Victorio Puche, María Garrido-Arandia, Laura Martin-Pedraza, Romero Sahagún Alejandro, José Damian López-Sánchez, Carmen Galán, Antonio Marin, Mayte Villaba, Araceli Díaz-Perales, Jose Antonio Cornejo, Maria Luisa Somoza

Natalia Perez-Sanchez should be included in the author byline. Natalia Perez-Sanchez should be listed as the first author, and affiliated with Allergy Clinical Unit, Hospital Regional Universitario de Málaga, Málaga-IBIMA. The contributions of this author are as follows: Formulated research goals and objectives, managed research data, conducted research and investigation, developed software solutions, led research planning and execution, visualized presentation and wrote the review and revision. The correct citation is: Perez-Sanchez N, Blanca M, Victorio Puche L, Garrido-Arandia M, Martin-Pedraza L, Romero Sahagún A, et al. (2020) Pru p 9, a new allergen eliciting respiratory symptoms in subjects sensitized to peach tree pollen. PLOS ONE 15(3): e0230010. https://doi.org/10.1371/journal.pone.0230010.

Jose Antonio Cornejo is not included in the author byline. Jose Antonio Cornejo should be listed as the 12^th^ author and affiliated with Allergy Research Group, Instituto de Investigación Biomédica de Málaga-IBIMA, Málaga, Spain. The contributions of this author are as follows: Formulated research goals and objectives, applied statistical, mathematical, computational to analyze data, developed design of methodology and software solutions, verified the overall replication of results and wrote the initial draft.

The following information is missing from the Funding statement: Grant PI15/01317, funded by Instituto de Salud Carlos III, Spain, and supported in part by the National Institutes of Health (NIH), USA.
