# Supplementary figures and images for: Pru p 9, a new allergen eliciting respiratory symptoms in subjects sensitized to peach tree pollen
Source: PLoS One. 2020 Mar 19;15(3):e0230010. doi: 10.1371/journal.pone.0230010 (PMC7082028; doi:10.1371/journal.pone.0230010)

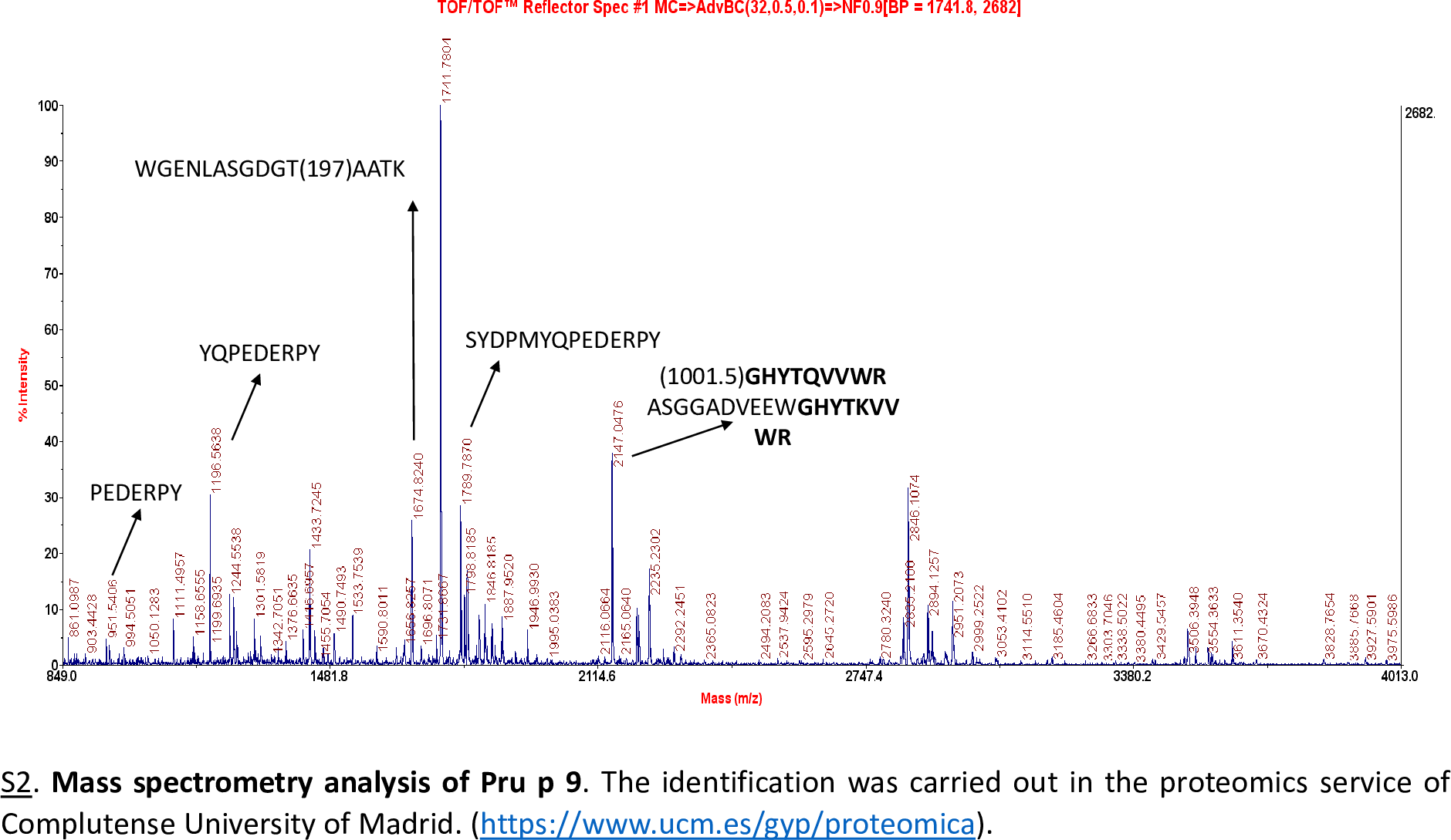

Supplement: S1 Fig — The identification was carried out in the proteomics service of Complutense University of Madrid. (https://www.ucm.es/gyp/proteomica). (TIF) [file pone.0230010.s001.tif]

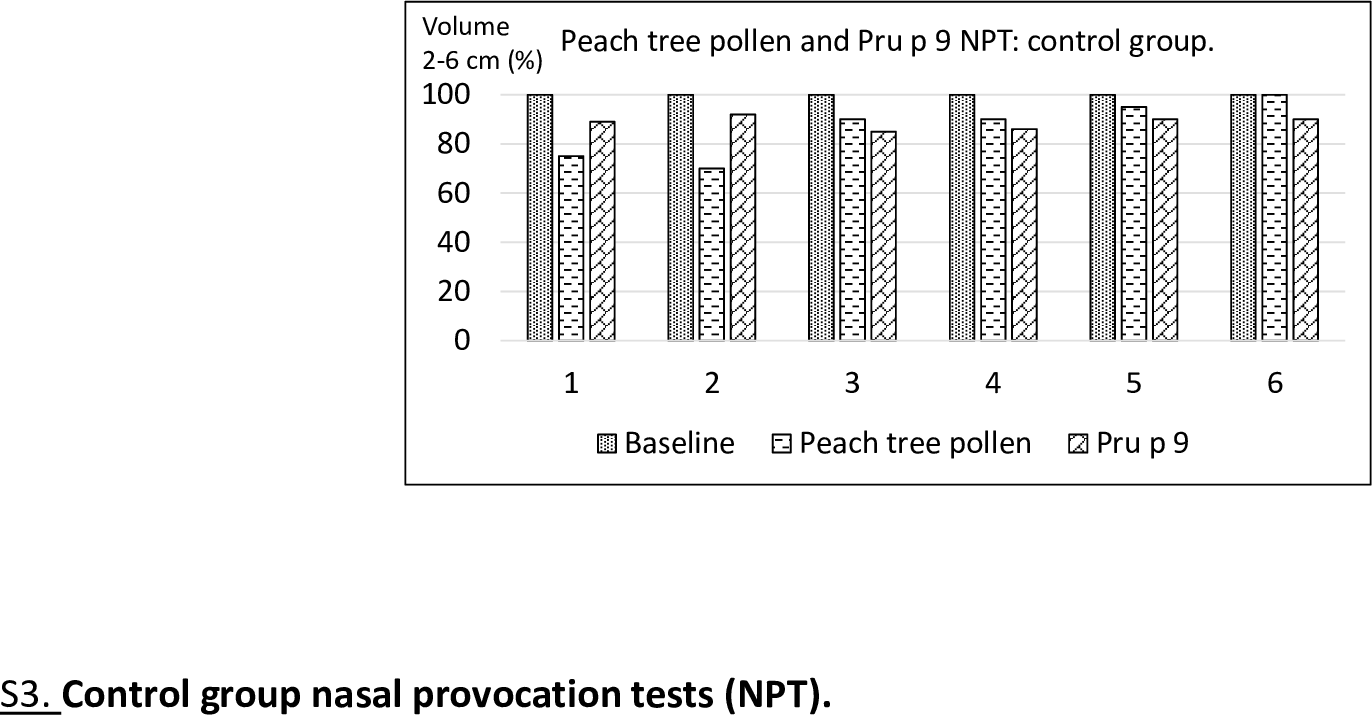

Supplement: S2 Fig — (TIF) [file pone.0230010.s002.tif]
