# Supplementary material for: Pru p 9, a new allergen eliciting respiratory symptoms in subjects sensitized to peach tree pollen
Source: PLoS One. 2020 Mar 19;15(3):e0230010. doi: 10.1371/journal.pone.0230010 (PMC7082028; doi:10.1371/journal.pone.0230010)
Supplement: S1 Raw images — (PDF) [file pone.0230010.s003.pdf]

The immunoblotting signal was developed by the ECL-Western Blotting reagent, and detected in a luminescent imager analyzer LAS3000 after 10 min. Quantitation of the signal was performed using the computer program Multigauge V3.0.

CBS signal was obtained using Coomassie Blue Staining R-250.

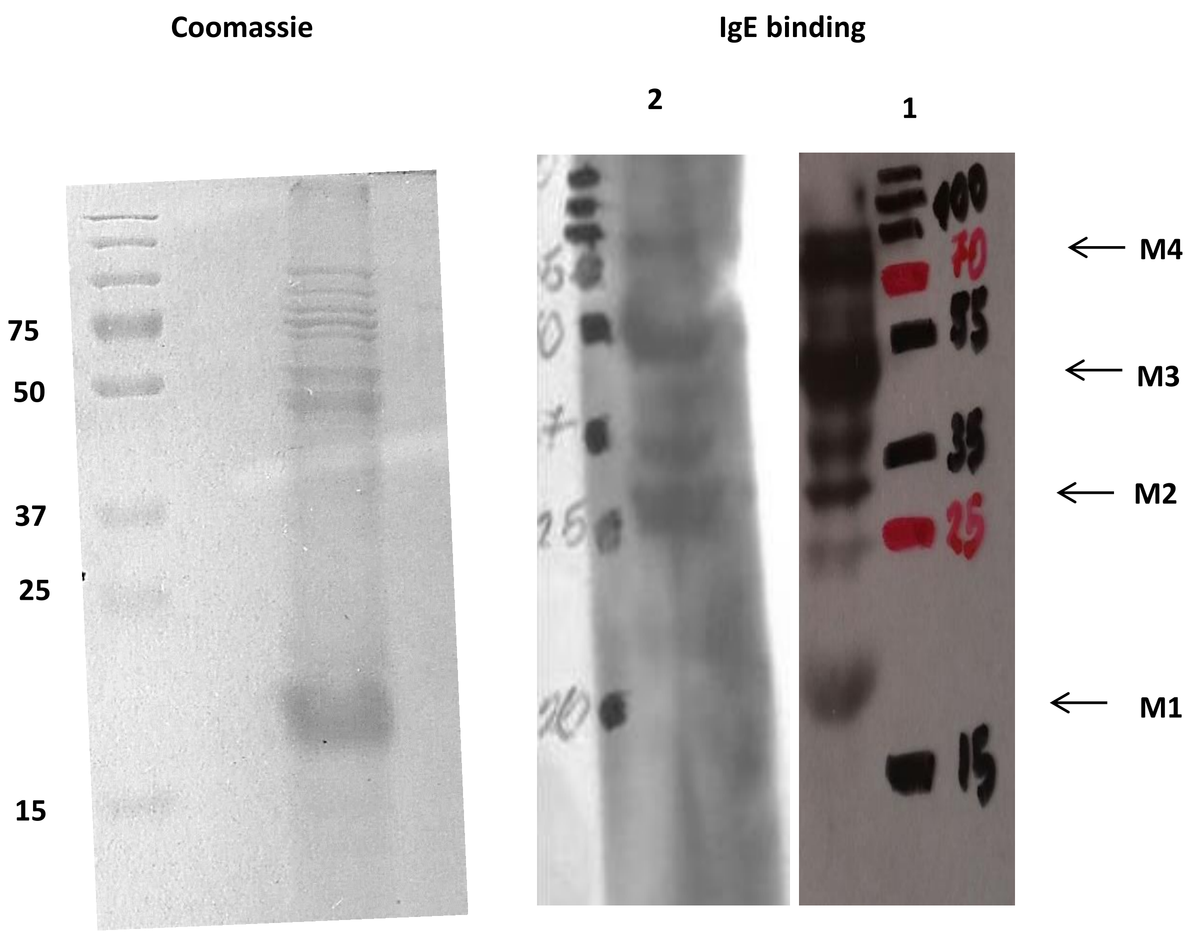

1: peach tree pollen allergic subjects
2: pollen allergic subjects.
M1: Pathogenesis related protein 1A.
M2: glucan endo-1,3-beta-glucosidase-like.
M3: Polygalacturonase.
M4: UTP-glucose-1-phosphate uridylyltransferase.

Figure 1.

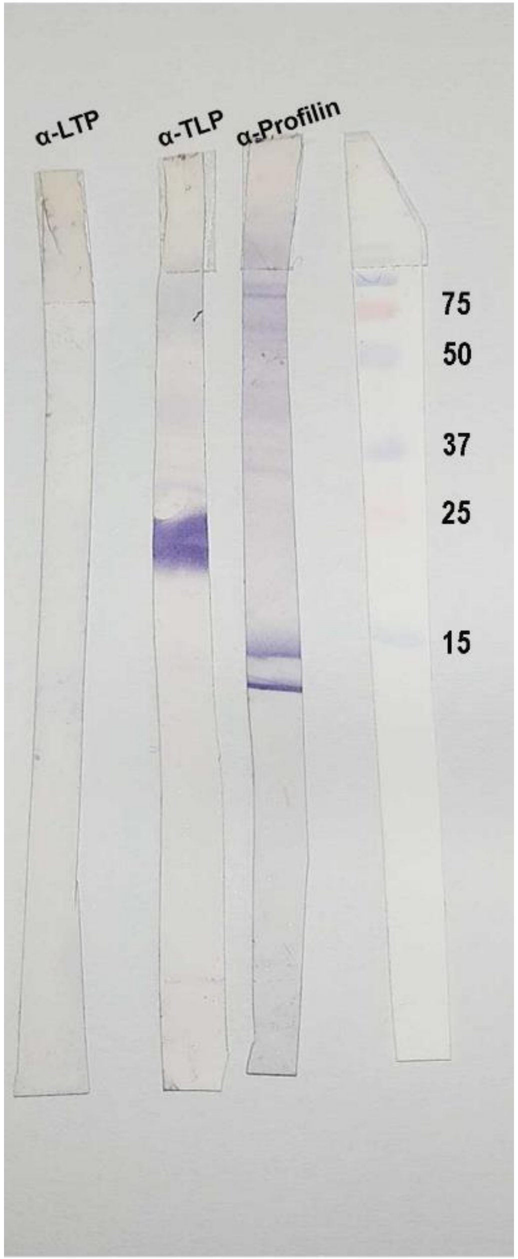

Figure 2.

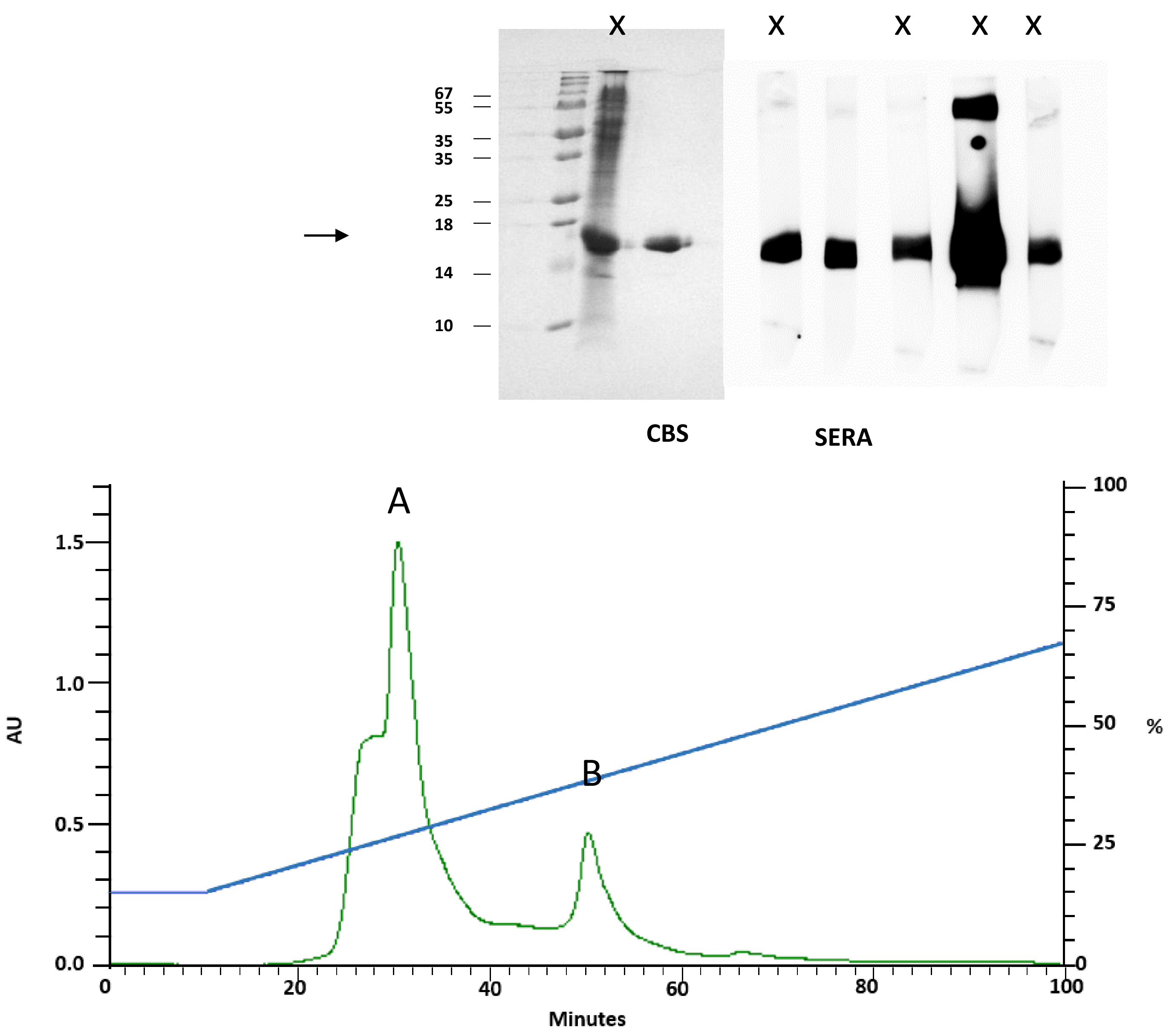

X
X
X
X
X
67
55
35
35
25
18
14
10
CBS
SERA
A
B
1.5
1.0
0.5
0.0
AU
0
20
40
60
80
100
Minutes
100
75
50
25
0
%

Figure 3.

Skin prick test (SPT) + to Peach tree pollen

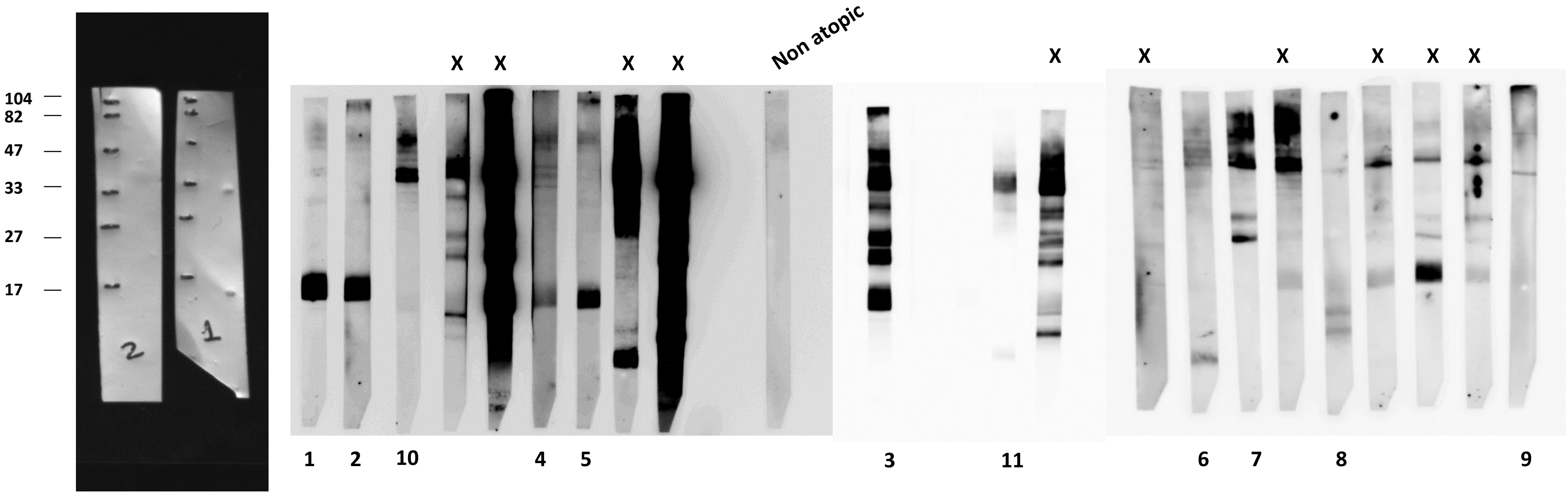

Figure 4.
