# Supplementary material for: Pru p 9, a new allergen eliciting respiratory symptoms in subjects sensitized to peach tree pollen
Source: PLoS One. 2020 Mar 19;15(3):e0230010. doi: 10.1371/journal.pone.0230010 (PMC7082028; doi:10.1371/journal.pone.0230010)
Supplement: Attachment — Submitted filename: Response to reviewers.docx [file pone.0230010.s004.docx]

PONE-D-19-19788
Pru p 9, a new allergen eliciting respiratory symptoms in subjects sensitized to Peach tree pollen.
PLOS ONE

**Journal requirements:**

**When submitting your revision, we need you to address these additional requirements.**

**1. Please ensure that your manuscript meets PLOS ONE's style requirements, including those for file naming. The PLOS ONE style templates can be found at
http://www.journals.plos.org/plosone/s/file?id=wjVg/PLOSOne_formatting_sample_main_body.pdf and http://www.journals.plos.org/plosone/s/file?id=ba62/PLOSOne_formatting_sample_title_authors_affiliations.pdf**

Response: This has been changed accordingly in the “Revised manuscript”

**2. Thank you for including your ethics statement: "The study was approved by our institutional Ethics Committee. All the participants have signed an informed consent."**

**a. Please amend your current ethics statement to include the full name of the ethics committee/institutional review board(s) that approved your specific study.**

Response: This has been amended.

**b. Once you have amended this/these statement(s) in the Methods section of the manuscript, please add the same text to the “Ethics Statement” field of the submission form (via “Edit Submission”).
For additional information about PLOS ONE ethical requirements for human subjects research, please refer to** [**http://journals.plos.org/plosone/s/submission-guidelines#loc-human-subjects-research**](http://journals.plos.org/plosone/s/submission-guidelines#loc-human-subjects-research)

Response: This has been made.

**3. Please provide additional details regarding participant consent. In the Methods section, please ensure that you have specified (1) whether consent was informed and (2) what type you obtained (for instance, written or verbal). If your study included minors, state whether you obtained consent from parents or guardians. If the need for consent was waived by the ethics committee, please include this information.**

Response: This has been added.

**4. Thank you for your Financial disclosure statement "Supported by grant FIS PI17/00615, RIRAAF RD12/0013/0005 and ARADyAL RD16/0006/0024/ RD16/0006/0014."**

**Please expand the acronyms so that it states the name of your funders in full.**

**This information should be included in your cover letter; we will change the online submission form on your behalf.**

Response: This has been expanded and included in the cover letter.

**5. PLOS ONE now requires that authors provide the original uncropped and unadjusted images underlying all blot or gel results reported in a submission’s figures or Supporting Information files. This policy and the journal’s other requirements for blot/gel reporting and figure preparation are described in detail at https://journals.plos.org/plosone/s/figures#loc-blot-and-gel-reporting-requirements and https://journals.plos.org/plosone/s/figures#loc-preparing-figures-from-image-files. When you submit your revised manuscript, please ensure that your figures adhere fully to these guidelines and provide the original underlying images for all blot or gel data reported in your submission. See the following link for instructions on providing the original image data: https://journals.plos.org/plosone/s/figures#loc-original-images-for-blots-and-gels.
 
In your cover letter, please note whether your blot/gel image data are in Supporting Information or posted at a public data repository, provide the repository URL if relevant, and provide specific details as to which raw blot/gel images, if any, are not available. Email us at plosone@plos.org if you have any questions.**

Response: The original blot/gel image data have been added as Supporting Information and noted in the cover letter.

**Additional Editor Comments (if provided):

The manuscript covers an interesting topic but prior to sending it for peer review it needs a major revision in the methods section. All the methods used and the recruitment proces for patients should be described in significantly more detail. Also there is a significant mistake in Table 2 regarding the significance of difference between 2 tested groups regarding clinical presentation (conjuctivitis, rhinitis, asthma).**

Response: In the “Revised manuscript” the methods section has been described precisely: methods used and recruitment of patients.

The mistake in Table 2 has been corrected. **[Note: HTML markup is below. Please do not edit.]

Reviewers' comments:


[NOTE: If reviewer comments were submitted as an attachment file, they will be attached to this email and accessible via the submission site. Please log into your account, locate the manuscript record, and check for the action link "View Attachments". If this link does not appear, there are no attachment files to be viewed.]


While revising your submission, please upload your figure files to the Preflight Analysis and Conversion Engine (PACE) digital diagnostic tool,** [**https://pacev2.apexcovantage.com/**](https://pacev2.apexcovantage.com/)**. PACE helps ensure that figures meet PLOS requirements. To use PACE, you must first register as a user. Registration is free. Then, login and navigate to the UPLOAD tab, where you will find detailed instructions on how to use the tool. If you encounter any issues or have any questions when using PACE, please email us at** [**figures@plos.org**](mailto:figures@plos.org)**. Please note that Supporting Information files do not need this step.**

Response: Our figure files have been uploaded to PACE.

**All the changes in the text have been highlighted in bold.**
