# Supplementary material for: Pru p 9, a new allergen eliciting respiratory symptoms in subjects sensitized to peach tree pollen
Source: PLoS One. 2020 Mar 19;15(3):e0230010. doi: 10.1371/journal.pone.0230010 (PMC7082028; doi:10.1371/journal.pone.0230010)
Supplement: Attachment — Submitted filename: Response to reviewers.docx [file pone.0230010.s005.docx]

**Review Comments to the Author**

**Reviewer #1:** 1. Summary and overall impression

The manuscript represents a comprehensive analysis of the etiology of the allergic sensitization in a population directly or indirectly exposed to peach tree allergens in the regions where this pollen is substantially cultivated. Importantly, it brings identification of allergen(s) responsible for pollen sensitization and respiratory allergic symptoms. Although the study has been performed using the renewed methods and does not utilize a new technology, it adds a great value to the field of allergy sensitization and atopy.

The Discussion contains a concise summary and relevance of the results. The results are extensively elaborated in the context of similar publications and explain how the obtained results fit into the other research groups' findings. The last paragraph should be extended regarding the impact on the future work, i.e. steps and ideas for further research should be proposed.

The manuscript is well structured and the scope of the project is clearly presented. It enables a reader a complete and easy progression through the work. A coherent presentation of the results is consequently easy to grasp. The accompanying figures are simple and illustrative.

**Response from the Authors:**

**The last paragraph has been extended with future work ideas (line 402-405).**

2. Evidence and examples:

Objective

The objective is nicely presented in a given theoretical background and argumented well enough to demonstrate its relevance and purpose. Given the size of the peach tree cultivated area and the prevalence of peach tree sensitization, the motivation is more than justified and the potential future application of the research is awaited.

Originality

The work uses established models of allergy study design and although it is not innovative, it builds up new knowledge to the topic.

Literature review

The literature review is nicely presented. The literature overview regarding the methodology of the study could be supported by a few more similar publications.

Proposed research

The proposed research is reasoned and well structured.

**Response from the Authors**:

**According to the suggestion, new references were added (22-24).**

Methodology

A major drawback in the experimental design is the lack of the control population group. The drawbacks in the methodology and similar methodology-related issues should be transparently declared.

**Response from the Authors**:

**A control group is now included: patients from an area where there are no peach tree cultivars. We performed skin prick test and nasal provocation test with peach tree pollen and Pru p 9. The results are highlighted in the text (line 188-190 Materials and Methods section and line 305-307 and 313-315 Results section) and a new figure was added (supplementary S3).**

**As mentioned in the previous version of the Manuscript, we used sera from subjects allergic to pollens but not to Pru p 9 as Pool 2 included in the text (line 101-103 Materials and Methods section) and Figure 1. These subjects did not recognize Pru p 9.**

**Moreover, we also performed immunoblotting with a non-atopic serum (line 295-296 Results section) as shown in Figure 4, which did not recognize any specific or unspecific band.**

Minor revisions:

Paragraph 2.3: Peach Tree Pollen protein extract and Pru p 9 purification from peach pollen

• In the methodology should be clearly stated that the two chromatographic steps were used to obtain purified Pru p 9. First step - ion exchange chromatography using Waters AccellTM Plus QMA Sep-PakR cartridge should also include the size of the pores of the used cartridge. The second step - reversed-phase chromatography is described in detail and clearly.

Paragraph: Analytical procedures: SDS-PAGE analysis.

**Response from the Authors**:

**This has been clarified in the text (line 110-117).**

• Tryptic digestion of bands excised from gel should include information regarding digestion mixture, concentration, period of incubation, temperature etc. or at least refer to a previous reference or established protocol. Although, in-gel trypsin digestion is a standard method it is relevant to state specific methodological details related to this work and type of sample.

• For MS analysis besides the type of used spectrometer for clarity the authors should add additional details related to the type of targeted plates, sample/matrix preparation (volume); type of laser with which is equipped mass spectrometer (wavelength, frequency). Need to add details regarding spectra recording: over which m/z range (linear mode?), at which accelerating voltage, average number of individual laser shots.

Optional additional figure: Mass determination spectra (figure of representative MALDI-ToF MS spectra to be added to the table with aminoacids residues verified by sequence analysis).

**Response from the Authors**:

**We have included a new section in Material and methods: Mass spectrometry analysis (line 123-155). In this new section we have explained in detail all the MS procedure including digestion mixture, concentration, period of incubation, temperature as well as sample/matrix preparation (volume) among others.**

**We have also added a supplementary figure (S2) with the Mass determination spectra of the PR-1a protein (Pru p 9) as an example.**

**Reviewer #2:**

1. More specific results and conclusions should be provided in the abstract.

**Response from the Authors:**

**The abstract has been extended with more results, also conclusions were added (line 30-33 and line 39-42).**

2. The criteria for patient selection and inclusion in the study need to be clarified, and indicate the number of respondents selected by which criteria

**Response from the Authors:**

**The criteria have been clarified and the number of the subjects has been added (line 72-77 Materials and Methods section, line 206-209 Results section).**

3. Provide a reference for the SPT, as well as a procedure for PT Pollen extracts.

**Response from the Authors:**

**The references for the SPT were added (References number 22, 23), the procedure for PT pollen extract clarified and a reference added (24).**

4. Clarify why respondents were done NPT

**Response from the Authors:**

**This is already explained in line 188-190. Nevertheless, the relevance of the reason why we did NPT with PT pollen and Pru p 9 is now better explained.**

5. How do you explain that exposure to a particular allergenic component of PT is manifested by symptoms due to sensitization to that component and not because of cross-reactivity, because you did not provoke with the cross-reactive allergens

**Response from the Authors:**

**We have included a control group of patients where PT pollen cultivars do not exist and data showed that although challenge with PT pollen could be positive, the nasal provocation test with Pru p 9 was negative. This data is included as Supplementary Figure (S3) and included in the text (line 188-190 Materials and Methods section and line 313-315 Results section).**

6. Indicate the most important weaknesses of your manuscript.

**Response from the authors:**

**This has been included in the last paragraph of the Discussion section (line 395-401).**
